# Spinal cord ependymomas and the appearance of other *de novo* tumors: a systematic review

**DOI:** 10.1186/1752-1947-8-438

**Published:** 2014-12-18

**Authors:** George Fotakopoulos, Konstantinos Vagkopoulos, Charalabos Gatos, Polikceni Kotlia, Alexandros Brotis

**Affiliations:** 36 Department of Neurosurgery, University Hospital of Thessaly, University Hospital of Larissa, Biopolis, 41110 Larissa, Thessaly Greece; 37 Department of Anesthesiology and Critical Care Medicine, University Hospital of Patras Medical School, Patras, Greece

**Keywords:** Another neoplasia, Secondary cancer, Spinal ependymoma

## Abstract

**Introduction:**

Ependymomas are rare glial tumors of the brain representing less than 5% of brain tumors. However, spinal cord ependymomas in adults account for over 60% of all ependymomas including those arising from the filum terminale and only 40% are intracranial. Reports of the appearance of another neoplasia at a different location in patients with spinal ependymoma are scarce.

**Methods:**

We searched PubMed for studies related to spinal cord ependymomas published over the last 30 years (from January 1984) and retrieved 1197.

**Results:**

We identified only two studies that met our criteria and we found an incidence of 9% of secondary neoplasias after treatment for spinal ependymoma. The neoplasms were diagnosed from 2 months to 20 years after patients underwent surgery for intraspinal ependymoma. These included pancreatic cancer, prostate cancer, Hodgkin lymphoma, intracranial meningioma, mucin-producing pulmonary adenocarcinoma, gastric cancer and astrocytoma.

**Conclusions:**

The genetic abnormalities affecting patients with spinal ependymomas may indicate a predisposition to the development of secondary cancers or a general failure of the repairing mechanism in their DNA. The unaffected survival rates in those individuals permit for a long period the accumulation of different mutations on the genome and thus the appearance of a second cancer. However, more studies are needed, particularly in young patients with high survival rates.

## Introduction

Ependymomas are rare glial tumors of the brain representing less than 5% of brain tumors [1–3]. However, spinal cord ependymomas in adults account for over 60% of all ependymomas including those arising from the filum terminale and only 40% are intracranial. According to the World Health Organization (WHO) classification, ependymomas are divided into three subtypes: subependymomas and myxopapillary ependymomas (MEPNs; WHO grade I), classic ependymomas (WHO grade II), and anaplastic ependymomas (WHO grade III). Compared with intracranial ependymomas, spinal ependymomas have a better prognosis [4] and gross total resection (GTR) is the gold standard in surgical treatment [5–9]. Thus they must be considered a unique clinical entity requiring specific management. MEPNs represent 13% of all ependymomas and are mainly found in the cauda equina, with occasional extension into the conus medullaris [10]. The remaining spinal cord ependymomas generally consist of the classic ependymoma type, which are primarily located in the cervical and thoracic regions [11].

Although cerebrospinal fluid metastasis occurs in 7% of patients with spinal ependymomas [12], multiple primary tumors in the central nervous system of different histological cell types are rare and more unusual seems to be the incidence of the development of a second cancer. This observation has not been reported in other tumor types and the mechanism still remains unclear. The purpose of the present study was to investigate the development of the second cancer in patients treated for spinal cord ependymomas.

## Results

After investigation of 1197 articles, only two articles were eligible [13, 14]. There were 100 patients surgically treated for intraspinal ependymomas [13, 14]. The average age at diagnosis was 41.67±18 years (range, 7 to 79 years) with a male-to-female ratio of 1.12:0.88. The study included two children. The most common presenting symptoms were local back pain (69.5%), radicular pain (70.5%) and urinary incontinence (19%). Mean time from symptom occurrence to diagnosis was 18.7 months (range, 1 month to 20 years). Mean follow-up time was 71.5 months (range, 2 months to 21 years). At primary surgery, the histological classification of tumors was as follows: MEPN (WHO grade I), 46 patients (46%); ependymoma (WHO grade II), 52 patients (52%); and anaplastic ependymoma (WHO grade III), two patients (2%). The tumors were located in the thoracic segment (n=16), cervical segment (n=18), conus medullaris (n=30), cauda equina (n=6), and filum terminale (n=28; Table 1). All tumors were operated through a posterior approach. GTR was performed in 73 patients (73%), subtotal resection in 26 patients (26%) and one patient (1%) underwent biopsy. Sixteen patients received radiotherapy after GTR (16/73 or 21.91%), one of these developed local recurrence and in three cases tumors scattered or metastasized. Six patients had a cerebrospinal fluid leak after surgery. Within the follow-up period, tumor recurrence was 8.21% (6/73 cases) in those patients who had undergone GTR. No association was found between age, tumor location, Ki-67 index, and recurrence of disease.

**Table 1:** **Baseline characterictics of patients**

Patients’ characteristics	Number	%
**Female**	47	47
**Location**		
1. Cervical	18	18
2. Thoracic	16	16
3. Conus medullaris	30	30
4. Filum terminale/cauda equine	36	36
**Histology**		
1. Myxopapillary ependymoma (grade I)	49	49
2. Ependymoma (grade II)	48	48
3. Anaplastic ependymoma (grade III)	3	3
**Secondary cancer**		
1. Gastric cancer	2	2
2. Lung cancer	2	2
3. Astrocytoma	1	1
4. Prostatic cancer	1	1
5. Pancreatic cancer	1	1
6. Hodgkin lymphoma	1	1
7. Intracranial meningioma	1	1

Secondary cancer in both studies was found in nine patients out of 100 suggesting that 9% of patients might develop a secondary cancer. The neoplasms were diagnosed 2 months to 20 years after patients underwent surgery for intraspinal ependymoma. These included pancreatic cancer, prostate cancer, Hodgkin lymphoma, intracranial meningioma, mucin-producing pulmonary adenocarcinoma, gastric cancer and astrocytoma (Table 1). In addition, only one patient (who later developed intracranial meningioma) had received radiotherapy, and the neoplasm was outside the radiation field. The remaining eight patients who developed a second cancer did not receive radiation therapy.

## Discussion

After performing a study on spinal ependymomas we found an increased incidence of secondary cancer [14]. Based on that, we set out to investigate whether other studies assessed the occurrence of secondary cancer. Secondary cancer in this review study was found in nine patients out of 100 who underwent surgery for spinal ependymoma, suggesting that 9% of patients might develop a *de novo* tumor.

There are reports supporting the view that gene mutations at different loci on chromosome 22 are associated with the pathogenesis of spinal ependymomas [15–17]. Several cytogenetic alterations, including deletions and translocations, constitute the most frequent changes in this chromosomal region [15, 18–20]. In addition, the contribution of other gene mutations on 17p13 and 10q25–26 loci have been projected as an alternative mechanism for the pathogenesis of spinal ependymomas [21]. Thus, all the above might suggest that in patients with spinal ependymomas the genome in some way is altered. This combined with the fact that the survival in these patients is not affected, may give rise to a continuous accumulation of different gene abnormalities which are more predisposed to the development of secondary cancers. The role of molecular imaging by positron emission tomography in those patients is very important, and may help to detect a secondary cancer and thus gives an opportunity for early diagnosis and a better outcome [22]. So we believe that this may be the reason why patients with spinal ependymomas might develop secondary neoplasia.

Radiotherapy is known to induce cancer. But this is not the fact in the present case because within the patients who developed secondary cancer only one was irradiated and the radiation field was outside the location of the second malignancy.

## Conclusions

The genetic abnormalities affecting patients with spinal ependymomas may indicate a predisposition to the development of secondary cancers or a general failure of the repairing mechanism in their DNA. The unaffected survival rates in those individuals permit for a long period the accumulation of different mutations on the genome and thus the appearance of a second cancer. However, more studies are needed, particularly in young patients with high survival rates.

## Methods

### Search strategy for identification of studies

PubMed searches were performed using a wide array of terms pertinent to spinal cord ependymomas. The exact search was done using the term -spin* and ependymoma- (last updated on 12 July 2014). We included studies published over the last 30 years (from January 1984). The reference lists of eligible articles and pertinent reviews were scrutinized. Two independent investigators for eligibility evaluated retrieved articles and disagreements were solved by consensus after discussion with a third investigator.

### Data extraction and definitions

This is a review study. From each eligible study we extracted the following information: author; journal; year; design; age of the study population; racial descent; whether analyses had been adjusted for multiple comparisons; whether analyses were acknowledged to be post hoc; and details on the definitions of all reported analyses.

From the 1197 related articles retrieved from the PubMed search, we excluded case reports analyzing less than four cases; any study involving children; non-English speaking publications and reports not related to humans; intracranial ependymomas; and any study with less than four cases with spinal ependymomas. Information was captured on all analyses performed and reported in any format and in any level of detail in the text, figures, tables, or supplementary material.

## References

[1] Chamberlain MC: **Ependymomas.** *Curr Neurol Neurosci Rep* 2003, **3:**193–199. 10.1007/s11910-003-0078-x 10.1007/s11910-003-0078-x 12691623

[2] Gilbert MR, Ruda R, Soffietti R: **Ependymomas in adults.** *Curr Neurol Neurosci Rep* 2010, **10:**240–247. 10.1007/s11910-010-0109-3 10.1007/s11910-010-0109-3 20425040

[3] Tseng JH, Tseng MY: **Survival analysis of 459 adult patients with primary spinal cancer in England and Wales: a population-based study.** *Surg Neurol* 2007, **67:**53–58. 10.1016/j.surneu.2006.04.011 10.1016/j.surneu.2006.04.011 17210300

[4] Henson JW: **Spinal cord gliomas.** *Curr Opin Neurol* 2001, **14:**679–682. 10.1097/00019052-200112000-00001 10.1097/00019052-200112000-00001 11723373

[5] Aghakhani N, David P, Parker F, Lacroix C, Benoudiba F, Tadie M: **Intramedullary spinal ependymomas: analysis of a consecutive series of 82 adult cases with particular attention to patients with no preoperative neurological deficit.** *Neurosurgery* 2008, **62:**1279–1285. 10.1227/01.neu.0000333299.26566.15 10.1227/01.neu.0000333299.26566.15 18824994

[6] Chang UK, Choe WJ, Chung SK, Chung CK, Kim HJ: **Surgical outcome and prognostic factors of spinal intramedullary ependymomas in adults.** *J Neurooncol* 2002, **57:**133–139. 10.1023/A:1015789009058 10.1023/a:1015789009058 12125974

[7] Epstein FJ, Farmer JP, Freed D: **Adult intramedullary spinal cord ependymomas: the result of surgery in 38 patients.** *J Neurosurg* 1993, **79:**204–209. 10.3171/jns.1993.79.2.0204 10.3171/jns.1993.79.2.0204 8331401

[8] Lin YH, Huang CI, Wong TT, Chen MH, Shiau CY, Wang LW, Ming-Tak Ho D, Yen SH: **Treatment of spinal cord ependymomas by surgery with or without postoperative radiotherapy.** *J Neurooncol* 2005, **71:**205–210. 10.1007/s11060-004-1386-y 10.1007/s11060-004-1386-y 15690140

[9] Kaner T, Sasani M, Oktenoglu T, Solmaz B, Sarloglu AC, Ozer AF: **Clinical analysis of 21 cases of spinal cord ependymoma : positive clinical results of gross total resection.** *J Korean Neurosurg Soc* 2010, **47:**102–106. 10.3340/jkns.2010.47.2.102 10.3340/jkns.2010.47.2.102 PMC2836443 20224707

[10] Rudà R, Gilbert M, Soffietti R: **Ependymomas of the adult: molecular biology and treatment.** *Curr Opin Neurol* 2008, **21:**754–761. 10.1097/WCO.0b013e328317efe8 10.1097/WCO.0b013e328317efe8 18989122

[11] Yang I, Nagasawa DT, Kim W, Spasic M, Trang A, Lu DC, Martin NA: **Chromosomal anomalies and prognostic markers for intracranial and spinal ependymomas.** *J Clin Neurosci* 2012, **19:**779–785. 10.1016/j.jocn.2011.11.004 10.1016/j.jocn.2011.11.004 PMC3615711 22516549

[12] Peschel RE, Kapp DS, Cardinale F, Manuelidis EE: **Ependymomas of the spinal cord.** *Int J Radiat Oncol Biol Phys* 1983, **9:**1093–1096. 10.1016/0360-3016(83)90402-9 10.1016/0360-3016(83)90402-9 6863076

[13] Halvorsen CM, Kolstad F, Hald J, Johannesen TB, Krossnes BK, Langmoen IA, Lied B, Rønning P, Skaar S, Spetalen S, Helseth E: **Long-term outcome after resection of intraspinal ependymomas: report of 86 consecutive cases.** *Neurosurgery* 2010, **67:**1622–1631. 10.1227/NEU.0b013e3181f96d41 10.1227/NEU.0b013e3181f96d41 21107192

[14] Voulgaris S, Alexiou GA, Zigouris A, Fotakopoulos G, Michos E, Katsiafas I, Savvanis G, Pachatouridis D: **Spinal ependymomas: prognostic factors and treatment results.** *J Cancer Res Ther* 2013, **9:**60–63. 10.4103/0973-1482.110371 10.4103/0973-1482.110371 23575076

[15] Ransom DT, Ritland SR, Kimmel DW, Moertel CA, Dahl RJ, Scheithauer BW, Kelly PJ, Jenkins RB: **Cytogenetic and loss of heterozygosity studies in ependymomas, pilocytic astrocytomas, and oligodendrogliomas.** *Genes Chromosomes Cancer* 1992, **5:**348–356. 10.1002/gcc.2870050411 10.1002/gcc.2870050411 1283324

[16] Sainati L, Montaldi A, Putti MC, Giangaspero F, Rigobello L, Stella M, Zanesco L, Basso G: **Cytogenetic t(11;17)(q13;q21) in a pediatric ependymoma. Is 11q13 a recurring breakpoint in ependymomas?** *Cancer Genet Cytogenet* 1992, **59:**213–216. 10.1016/0165-4608(92)90218-W 10.1016/0165-4608(92)90218-w 1581886

[17] Griffin CA, Long PP, Carson BS, Brem H: **Chromosome abnormalities in low-grade central nervous system tumors.** *Cancer Genet Cytogenet* 1992, **60:**67–73. 10.1016/0165-4608(92)90235-Z 10.1016/0165-4608(92)90235-z 1591709

[18] Bijlsma EK, Voesten AM, Bijleveld EH, Troost D, Westerveld A, Mérel P, Hulsebos TJ: **Molecular analysis of genetic changes in ependymomas.** *Genes Chromosomes Cancer* 1995, **13:**272–277. 10.1002/gcc.2870130407 10.1002/gcc.2870130407 7547635

[19] Weremowicz S, Kupsky WJ, Morton CC, Fletcher JA: **Cytogenetic evidence for a chromosome 22 tumor suppressor gene in ependymoma.** *Cancer Genet Cytogenet* 1992, **61:**193–196. 10.1016/0165-4608(92)90085-M 10.1016/0165-4608(92)90085-m 1638502

[20] Wernicke C, Thiel G, Lozanova T, Vogel S, Kintzel D, Jänisch W, Lehmann K, Witkowski R: **Involvement of chromosome 22 in ependymomas.** *Cancer Genet Cytogenet* 1995, **79:**173–176. 10.1016/0165-4608(94)00148-5 10.1016/0165-4608(94)00148-5 7889515

[21] Yokota T, Tachizawa T, Fukino K, Teramoto A, Kouno J, Matsumoto K, Emi M: **A family with spinal anaplastic ependymoma: evidence of loss of chromosome 22q in tumor.** *J Hum Genet* 2003, **48:**598–602. 10.1007/s10038-003-0078-3 10.1007/s10038-003-0078-3 14566482

[22] Sandu N, Pöpperl G, Toubert ME, Spiriev T, Arasho B, Orabi M, Schaller B: **Current molecular imaging of spinal tumors in clinical practice.** *Mol Med* 2011,**17**(3–4)**:**308–316. 10.2119/molmed.2010.00218 PMC3060992 21210073

